# Knowledge, Attitude, and Practices Related to Cervical Cancer Among Adult Women in Azad Kashmir: A Hospital-based Cross-sectional Study

**DOI:** 10.7759/cureus.4234

**Published:** 2019-03-11

**Authors:** Arslaan Javaeed, Sana Shoukat, Saddaf Hina, Zartasha Hameed, Sanniya Khan Ghauri, Malik Mahmood Ahmed

**Affiliations:** 1 Pathology, Poonch Medical College, Rawalakot, PAK; 2 Pathology, Sheikh Khalifa Bin Zayed Hospital, Rawalakot, PAK; 3 Emergency Medicine, Shifa International Hospital, Islamabad, PAK; 4 Pathology, Azad Jammu Kashmir Medical College, Muzzfarabad, PAK

**Keywords:** cervical cancer, pap smear, screening, adult women, pakistan

## Abstract

Objective

To assess the knowledge, attitude, and practices (KAP) related to cervical cancer among the adult women of Azad Kashmir, Pakistan.

Methods

A cross-sectional study, involving 594 patients visiting the Gynecology and Obstetrics outpatient departments of Khalifa bin Zayed Hospital, Rawalakot, Azad Kashmir, Pakistan, was done. The study questionnaire (interviewer-administered) included 26 items to measure the knowledge, attitude, and practices related to cervical cancer and was formulated and validated with the help of gynecologists and epidemiologists. Descriptive statistics were used to present the knowledge, attitude, and practice level of respondents. The respondents’ knowledge, attitude, and practice score was compared across gender and level of education. Data analysis was done using SPSS v 23.0 (IBM Corporation, Armonk, NY, US) at 95% CI.

Results

A total of 346 (58.2%) women heard about cervical cancer and 210 (35.4%) women heard about the pap smear test. Thirty-five women (5.9%) underwent a pap smear test in their lifetime. More than half (51.7%) thought that undergoing a pap smear test is embarrassing. But 382 respondents (64.3%) will undergo a pap smear test if the test is provided free of cost. Unmarried women had a better KAP score as compared to married women (13.58±5.14 vs 9.12±4.04, p<.001). The KAP score was significantly different in respondents with different levels of education (p<.001).

Conclusion

This study showed a better KAP score as compared to previous Pakistani studies but, still, there is plenty of room to improve. Women of developed countries have significantly better knowledge, attitude, and practices related to cervical cancer. Local authorities may run a free pap smear screening program in communities to detect cervical cancer early.

## Introduction

Cervical cancer is the second most common cancer among women worldwide [1]. Eighty-six percent of all cervical cancer diagnosed and 88% of death due to cervical cancer occur in developing regions of the world [1-2]. Worldwide, there were approximately 493,243 new cases and 273,505 deaths attributed to cervical cancer in 2002, which is about one-tenth of total female cancer deaths [3]. The present exact rate of the incidence and prevalence of cervical cancer is not known in Pakistan because it is an ignored disease in terms of screening and prevention [4]. In 2002, the prevalence of cervical cancer in Pakistani women was 0.009% while in 2008, it was 0.019%, according to advance research started by the World Health Organization (WHO) [5].

As Pakistan is a developing country, human papillomavirus (HPV) is a major threat to public health. To date, HPV screening is generally not implemented in Pakistan. A major hurdle to the exact statistical assessment and evaluation of the HPV epidemic are social restrictions [6]. In Pakistan, to date, it is a taboo to discuss sexually transmitted diseases and sexual education, due to which most of the female population, mainly from the rural areas, have a poor understanding of sexually transmitted diseases (STDs) and gender-specific cancers. Perhaps due to this, cervical cancer caused by HPV is ranked the third major contributing source of deaths among women in Pakistan [7].

For establishing successful strategies and increasing the utilization of preventive services, there is a need to explore the extent to which the general female population is aware of cervical cancer-related problems. And there is scanty information about the knowledge and awareness of Pakistani women on this potential issue. In this hospital-based study, we aim to assess the knowledge, attitude, and practices related to cervical cancer among adult Pakistani women.

## Materials and methods

A cross-sectional, interview-based study was conducted in the Gynecology and Obstetrics outpatient department (OPD), Sheikh Khalifa bin Zayed Hospital, Rawalkot, Azad Kashmir (CMH), from March to September 2018. As the current prevalence rate of cervical cancer in Pakistan was unknown, we calculated a minimum required sample size of 384 at the 95% confidence interval and prevalence of .05. In order to get the required sample size, we have invited all Gynecology and Obstetrics OPD patients during the study period. A total of 594 patients gave consent and fully answered the questionnaire. The study questionnaire was developed with the help of gynecologists and epidemiologists of the Sheikh Khalifa bin Zayed Hospital. The questionnaire was checked for internal consistency. The questionnaire included baseline characteristics, cervical cancer-related knowledge, attitude, and practice sections (26 items). The purpose of the study was clearly stated to the respondents, and consent was secured. Ethical approval was taken from the ethical review committee of Sheikh Khalifa bin Zayed Hospital, Rawalkot, Azad Kashmir, Pakistan.

Statistical analysis

Baseline characteristics and cervical cancer KAP questions were presented as frequencies and percentages. The number of correct responses from each respondent was counted. The mean number of correct responses was compared across gender and education level by the independent samples t-test and one-way analysis of variance (ANOVA), respectively. Data were presented in tables and charts.

The analysis was performed at the 95% confidence interval using the Statistical Package for Social Science (SPSS), version 23.0 (IBM, Armonk, NY, USA).

## Results

The interviewer approached 700 Gynecology and Obstetrics OPD patients (adult women) with the study questionnaire; out of these 594 women gave consent and completely answered all the questions (response rate 84.85%). The mean age of all respondent was 26.61±0.29 years. Almost half of the respondents, 288 (48.5%), were married and the rest were unmarried. The respondents’ level of education was as follows: no formal education 51 (8.6%), primary level 81 (13.6%), secondary level 78 (13.1%), higher secondary level 144 (24.2%), and graduate level 240 (40.4%).

More than one-third, 211 (35.5%), respondents never heard of cervical cancer. Most of the women did not know about any of the symptoms of cervical cancer. More than half, 307 (51.7), thought that going for the pap smear test was embarrassing. Cervical cancer knowledge and attitude related to the three-point Likert scale questions and responses are presented in Table 1.

**Table 1:** Responses to knowledge and attitude related to Likert-scale questions *Question nos. 15, 16, and practice-related questions (25 and 26) were not formulated on the Likert scale and were presented later.

Categories	Question Number	Question/statement	Yes	No	Don’t know
Knowledge	1	Have you ever heard of cervical cancer?	346 (58.2)	211 (35.5)	37 (6.2)
	2	Bleeding in between menstrual cycle (period) is a symptom	246 (41.4)	76 (12.8)	272 (45.8)
	3	Foul smelling discharge is a symptom	260 (43.8)	88 (14.8)	246 (41.4)
	4	Postmenopausal bleeding is a symptom	239 (40.2)	82 (13.8)	273 (46.0)
	5	Periods heavier and of longer duration than normal is a symptom	229 (38.6)	92 (15.5)	273 (46.0)
	6	HPV is a risk factor	242 (40.7)	84 (14.1)	268 (45.1)
	7	Multiple sexual partners are a risk factor	376 (63.3)	46 (7.7)	172 (29.0)
	8	Early age of coitus is a risk factor	260 (43.8)	84 (14.1)	250 (42.1)
	9	Tobacco smoking is a risk factor	345 (58.1)	90 (15.2)	159 (26.8)
	10	History of sexually transmitted disease is risk factor	378 (63.6)	64 (10.8)	152 (25.6)
	11	Poor menstrual hygiene is risk factor	425 (71.5)	48 (8.1)	121 (20.4)
	12	Prolonged use of birth control pills (>5) is a risk factor	362 (60.9)	76 (12.8)	156 (26.3)
	13	Multiple pregnancies are a risk factor	330 (55.6)	106 (17.8)	158 (26.6)
	14	Have you ever heard of pap smear test?	210 (35.4)	365 (61.4)	19 (3.2)
	17	Pap smear test is painful	140 (23.6)	66 (11.1)	388 (65.3)
Attitude	18	Intermenstrual bleed should be considered normal	87 (14.6)	469 (79.0)	38 (6.4)
	19	Going for pap smear test is embarrassing	307 (51.7)	194 (32.7)	93 (15.7)
	20	Going for pap smear test is expensive	162 (27.3)	89 (15.0)	343 (57.7)
	21	If you would be offered free cervical cancer screening, would you be willing to be screened?	382 (64.3)	155 (26.1)	57 (9.6)
	22	I am afraid of screening because of cancer detection	200 (33.7)	347 (58.4)	47 (7.9)
	23	If someone is suffering from cervical cancer, you will keep a distance from that lady	128 (21.5)	420 (70.7)	46 (7.7)
	24	Do you plan to be screened for cervical cancer in next 03 years?	153 (25.8)	280 (47.1)	161 (27.1)

**Figure 1:**
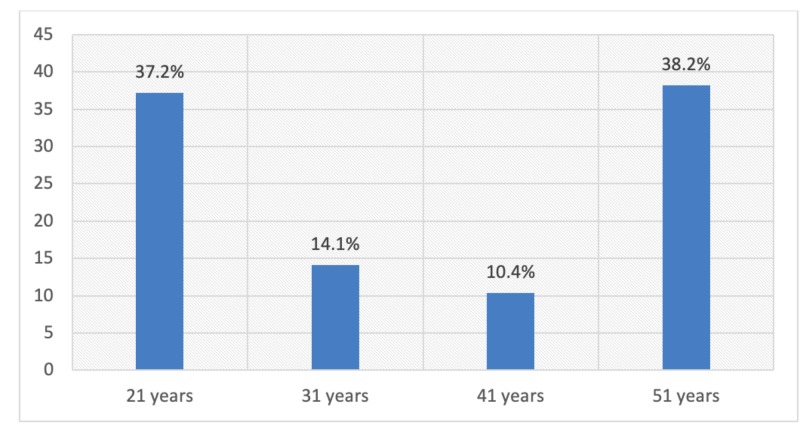
Distribution of respondents by knowledge regarding the correct age of starting a pap smear test

**Figure 2:**
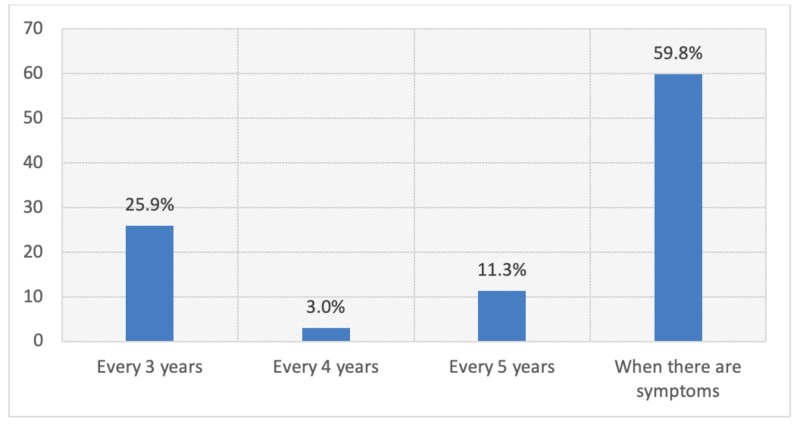
Distribution of respondents by knowledge regarding the frequency of the pap smear test

**Figure 3:**
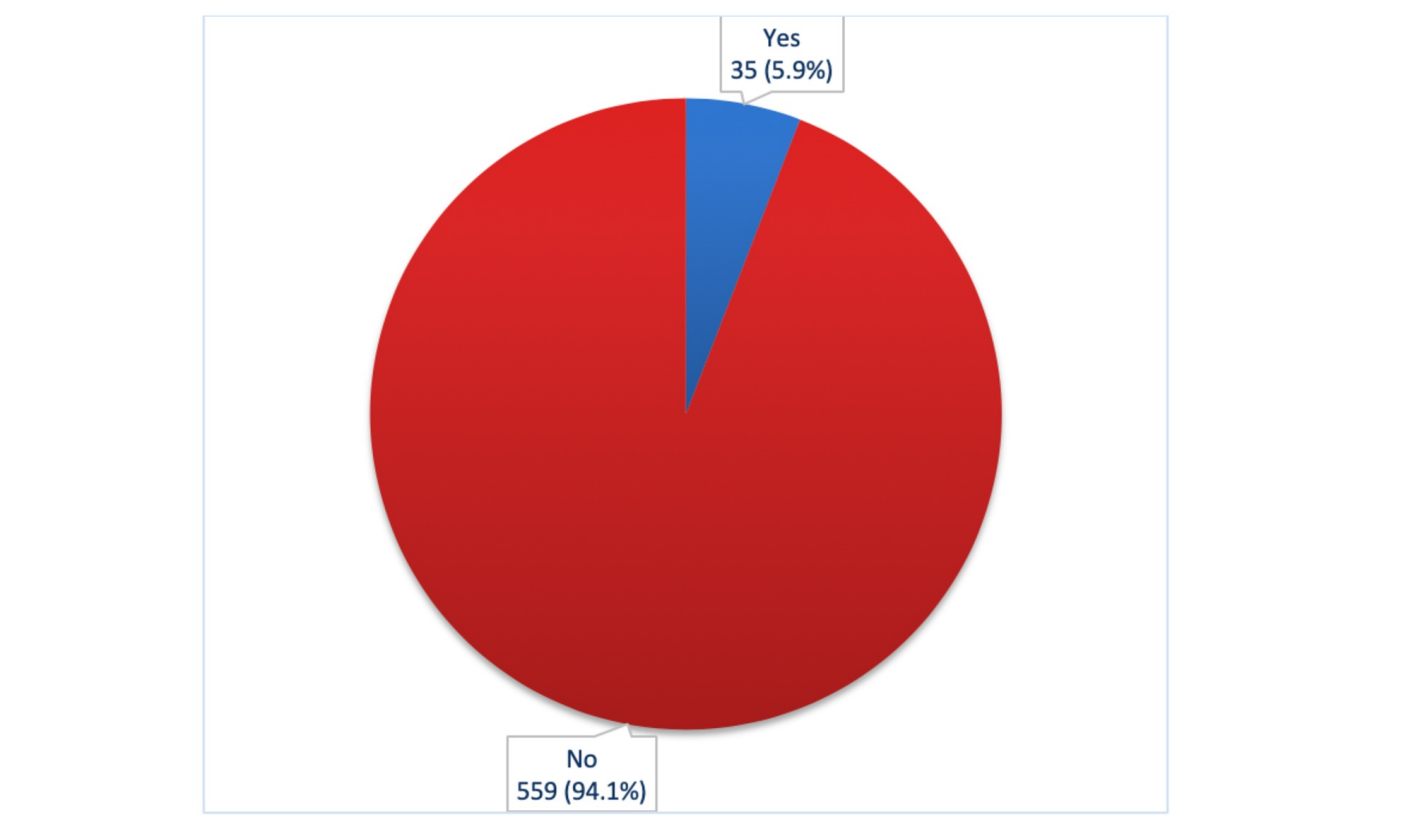
Distribution of respondents who underwent screening for cervical cancer

**Figure 4:**
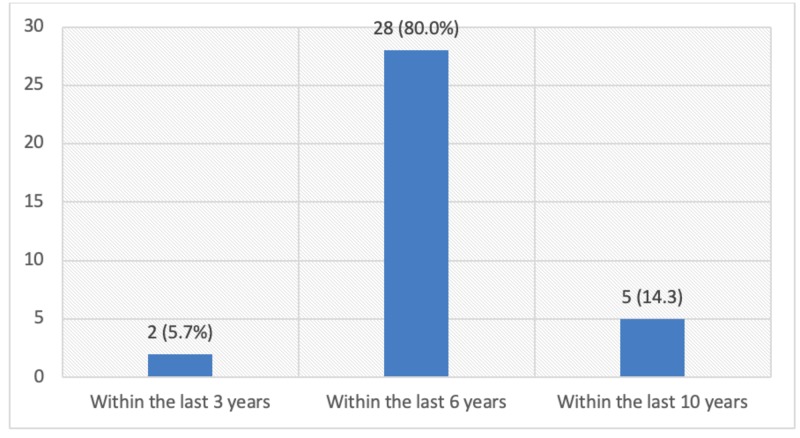
Distribution of respondents by the last time they were screened for cervical cancer (n=35)

Only 37.2% respondents knew the correct age of starting a pap smear test and 25.9% knew it should be done every three years. Only a handful of patients, 35 (5.9%), underwent screening for cervical cancer in their lifetime and among them, only two respondents underwent the test within the last three years, as shown in Figures 1-4.

Unmarried women had a better knowledge, attitude, and practice score as compared to married women (13.58±5.14 vs 9.12±4.04, p<.001). Women with different levels of education had statically significantly different knowledge, attitude, and practice scores (p<.001) (Tables 2-3).

**Table 2:** Comparison of knowledge, attitude, and practice scores between married and unmarried women

Marital status	N	Mean ± SD	p-value
Married	288	9.12 ± 4.04	< .001
Unmarried	306	13.58 ± 5.14	

**Table 3:** Comparison of knowledge, attitude, and practice scores among different levels of education

Level of education	N	Mean ± SD	p-value
No formal education	51	6.84 ± 2.98	< .001
Primary	81	9.17 ± 5.01	
Secondary	78	8.15 ± 4.02	
Higher secondary	144	13.85 ± 4.36	
Graduate and above	240	12.74 ± 4.89	

## Discussion

The current study identified inadequate knowledge, attitude, and practice regarding cervical cancer. Similar results were revealed by a study done in India, where 64% of the respondents knew about one or more symptoms of cervical cancer and 39% knew about the risk factors [8]. A Nigerian study also revealed low knowledge (43.5% respondents) of cervical cancer screening [9]. Similar studies done in other developing countries showed similar results. For example, an Ethiopian study showed 9.9% of the participants had undergone cervical cancer screening in their lifetime while a Cambodian study showed 34% women had heard of cervical cancer and 7% had a pap smear test [10-11]. Developing countries have a higher burden of cervical cancer (80% cases); poor knowledge, attitude, and practice may be a contributing factor in this scenario.

In the current study, we have observed higher knowledge among unmarried women as compared to married women. This might be due to the fact that unmarried women are relatively younger and have more interest in the potential source of information regarding cervical cancer, e.g. Internet and social media. Formal education may also play a role in increasing cervical cancer-related knowledge; this study revealed the lowest level of knowledge among those who had no formal education.

When comparing with the local (Pakistani) studies, we have found our current study showed slightly better knowledge and attitude among the respondents as compared to a previous study done in two public hospitals of Karachi, Pakistan [12]. Another previous Pakistani study found 2.6% of the respondents underwent a Pap smear test while the current study found 5.9% underwent the test [13]. This suggests that the knowledge, attitude, and practice of cervical cancer in Pakistan is improving with time. Several studies in developed countries have concluded that even in the higher socio-economic strata of South Asian women, the rates of Pap test receipt remain low due to a lack of awareness [14-16]. Therefore, improving the knowledge of the population regarding cervical cancer screening is one of the most important steps in enhancing the Pap test coverage among Pakistani women.

Limitation

Being a single center study, it may not involve the sample representative of the population of the whole country. The reason behind low knowledge, attitude, and practice could not be established, as it was a cross-sectional study.

Recommendation

Further, similar studies can be done to compare the results and understand the pattern of improvement of knowledge. Improvement in female education rates, open conferences regarding sexual health, and increasing information availability will eventually improve the attitude and practice toward cervical cancer screening.

## Conclusions

Overall poor knowledge, attitude, and practice regarding cervical cancer is observed all over the developing world. This study showed a slightly higher knowledge as compared to previous Pakistani studies. More than two-thirds of women will undergo a Pap smear test if the service is given free of cost. Local authorities may run free Pap smear test centers to detect cervical cancer early and lower the nationwide cervical cancer burden.
